# Screening of asymptomatic rheumatic heart disease among refugee/migrant children and youths in Italy

**DOI:** 10.1186/s12969-019-0314-9

**Published:** 2019-04-02

**Authors:** Fortunata Condemi, Gabriele Rossi, Miguel Lupiz, Antonio Pagano, Federica Zamatto, Stefano Marini, Francesco Romeo, Gianfranco De Maio

**Affiliations:** 1 Médecins Sans Frontières-Operational Centre Brussels, Italy Mission, Rome, Italy; 2 Department of Cardiovascular Disease, University of Rome Tor Vergata, Rome, Italy; 3 Médecins Sans Frontières-Operational Centre Brussels, Medical Department, Brussels, Belgium; 4 National Institute for Health, Migration and Poverty, Rome, Italy; 5 Médecins Sans Frontières-Operational Centre Brussels, Operations Department, Rome, Italy; 6 Department of Clinical Sciences and Translational Medicine, University of Rome Tor Vergata, Rome, Italy

**Keywords:** ‘definite Rheumatic Heart Disease’, ‘borderline Rheumatic Heart Disease’, Echocardiography, Migrants health, Population screening

## Abstract

**Background:**

Rheumatic heart disease (RHD) is a chronic condition responsible of congestive heart failure, stroke and arrhythmia. Almost eradicated in high-income countries (HIC), it persists in low- and middle-income countries. The purpose of the study was to assess the feasibility and meaningfulness of ultrasound-based RHD screening among the population of unaccompanied foreign minors in Italy and determine the burden of asymptomatic RHD among this discrete population.

**Methods:**

From February 2016 to January 2018, Médecins Sans Frontières conducted a weekly mobile screening by echocardiography in reception centers and family houses for unaccompanied foreign minors in Rome, followed by fix echocardiographic retesting for those resulting positive at screening. ‘Definite’ and ‘borderline’ cases were defined according to the World Hearth Federation criteria.

**Results:**

Six hundred fifty-three individuals (13–26 years old) were screened; 95.6% were below 18 years old (624/653). Six ‘definite RHD’ were identified at screening, yielding a detection rate of 9.2‰ (95% CI 4.1–20.3‰), while 285 (436.4‰) were defined as ‘borderline’ (95% CI 398.8–474.9‰). Out of 172 “non-negative borderline” cases available for being retested (113 “non-negative borderline” lost in follow-up), additional 11 were categorized as ‘definite RHD’, for a total of 17 ‘definite RHD’, yielding a final prevalence of 26.0‰ (95% CI 16.2–41.5‰) (17/653), and 122 (122/653) were confirmed as ‘borderline’ (final prevalence of 186.8‰, 95% CI 158.7–218.7). In multivariate logistic regression analysis the presence of systolic murmur was a strong predictor for both ‘borderline’ (OR 4.3 [2.8–6.5]) and ‘definite RHD’ (OR 5.2 [1.7–15.2]), while no specific country/geographic area of origin was statistically associated with an increased risk of latent, asymptomatic RHD.

**Conclusions:**

Screening for RHD among the unaccompanied migrant minors in Italy proved to be feasible. The burden of ‘definite RHD’ was similar to that identified in resource-poor settings, while the prevalence of ‘borderline’ cases was higher than reported in other studies. In view of these findings, the health system of high-income countries, hosting migrants and asylum seekers, are urged to adopt screening for RHD in particular among the silent and marginalized population of refugee and migrant children.

## Introduction

Rheumatic Heart Disease (RHD), a sequaela of Rheumatic Fever (RF) affecting the heart valve system, often evolves in congestive heart failure and arrhythmias, requiring surgery after a period of asymptomatic, latent phase (silent) [1]. It is reported that 320,000 RHD-related deaths occur annually [2]. Almost eradicated in high income countries (HICs) (eg: prevalence in the US is less than 0.05‰) [3], the disease persists in the middle and low income countries (MICs and LICs). With the introduction of ultrasound screening to detect early stage RHD [4], the estimate of the global burden of RHD has been recently revised at more than 33 million existing cases, which is higher than the previous conservative 15.6 to 19.6 million cases in 2005 [5–8].

Attempts to tackle the burden of RHD, spearheaded by WHO and World Heart Federation’s (WHF) [9, 10], have not been effectively implemented in LICs and MICs because of the structural weaknesses of their health systems: today, RHD remains still the main cause of cardiac-related mortality in poor countries [11].

On the other hand, while RHD and its socio-economic determinants are concentrated in low resource settings, it is useful to recognize that the recently intensified synergic phenomena of globalization, migration and refugee crises have displaced the common belief according to which RHD is just confined to developing nations. Rather, RHD has to be seen as a global health problem, requiring a global policy response [12, 13].

The current enormous refugee crisis worldwide and especially in Europe represents a paradigmatic translational shift. The health systems of the poor countries are not any longer left alone in bearing the burden of RHD, as wealthy countries are now called to deal, in a substantial way, with the poor people and their diseases [13, 14].

Refugees and displaced persons from settings affected by crisis often have complex needs and an increased risk of health problems related to their journeys. Many migrants experience lack of access to and continuity of health care. This makes particularly challenging to detect and manage asymptomatic, early stage RHD, which could prevent excessive RH-related morbidity and mortality. The few reports in the literature describing the ‘RHD and migrants’ topic refer to advanced-stage, severe, symptomatic RHD in need of cardiac surgery [15].

In response to the need of assessing the true prevalence of RHD among refugee and migrant populations in high resource setting [14, 16], Médecins Sans Frontières (MSF) [17], in collaboration with the National Institute for Health, Migration and Poverty (NIHMP) and expert operators from the Cardiology Unit of the Roma 2 University Hospital, conducted a 2-stage intervention (ultrasound-based screening and retesting) for the migrant youth population hosted in reception centers in the Municipality of Rome, Italy.

## Material and methods

**Fig. 1:**
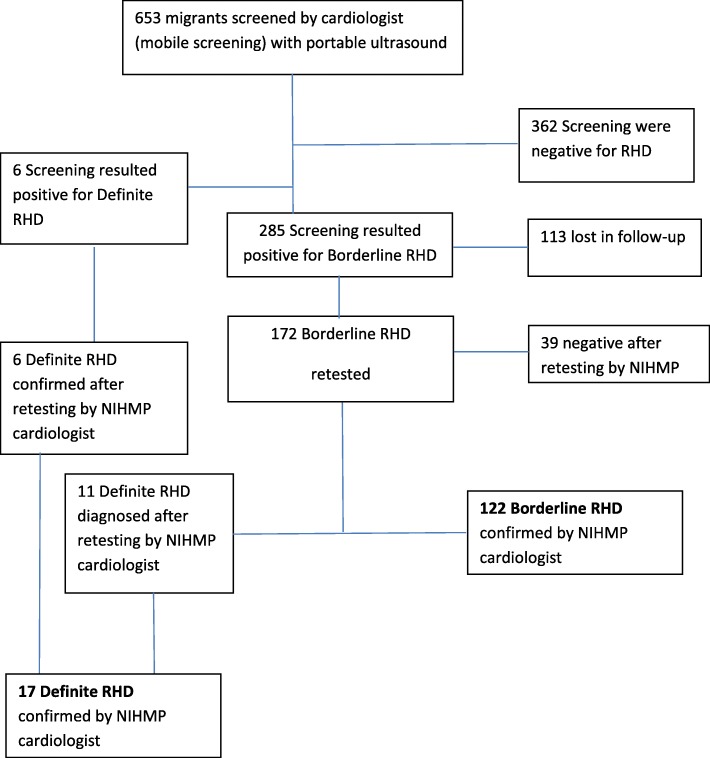
Flow diagram of the Screening and Retesting activity for RHD. The combined screening of 653 migrants/refugees children and youths, carried out through the two-dimensional echocardiogram “GE Healthcare 2007”, and the retesting activity performed with the fixed ultrasound echocardiography machine Siemens “Acuson X700”, allowed to identify 17 ‘definite RHD’ and 122 ‘borderline RDH’ for a final detection rate of 26.0‰ and 186.8‰, respectively

The strategy of this observational study revolved around the idea to carry out, in first instance, an echocardiographic screening through portable device among young people between 10 and 25 years, belonging to groups of migrants coming from endemic areas (Northern Africa, Western Sub-Saharan Africa, Eastern Sub-Saharan Africa and Southern Asia). The enrollment period for the screening spanned from February 2016 to January 2018, with a frequency of one to two weekly sessions (and an expected average of 8 echocardiographic tests per session). The decision to implement the intervention in reception centers of unaccompanied minors was informed by reasons of opportunity, as in Italy is possible to easily identify the target population of the study (migrant/refugee minors) in such protected locations managed by the public Authority. The study design also included a second, more accurate test for those individuals who resulted positive at the screening (Fig. 1). Symptomatic cases with signs of cardiac failure or impairment of the heart function were referred for further evaluation to the Cardiology Unit of the Rome 2 University Hospital.

### Study methodology and patient population characteristics

The exclusion criteria included a) age higher than 25 years, b) permanence less than 10 years in environment at high risk for streptococcal infection exposure (countries of origin or transit refugees’ camps); coexistence of congenital cardiac disease or other cardiac pathologies; previous diagnosis of RF/RHD treated with antibiotic therapy.

The mobile team, consisting of a cardiologist, a coordinating nurse and a cultural linguistic mediator, visited several reception centers for unaccompanied foreign minors managed by the Municipality of the city of Rome, according to a planned timetable. Participants were informed of the study and were asked to provide oral consent. Assent was obtained from minors > 13 years of age in the presence of adult witness, while for children < 13 years of age, oral consent was provided by the guardian of the minor. The included study subjects received a cardiologic consultation and an echocardiographic examination and each of them was given a medical card reporting the results of the consultation and examination, with indications for possible follow-up controls.

Previous clinical history was collected at the time of screening, along with information about previous throat infections and high fever, previous hospitalizations and ability to access the Italian health system. Screening was carried out through a two-dimensional echocardiogram using a portable machine GE Vivid e Cardiovascular Ultrasound System (GE Healthcare 2007) with transducer 3S-RS.

Guidelines of World Heart Federation (WHF) for the RHD echocardiographic were used [18] to differentiate the cases in ‘definite’, ‘borderline’ RHD and ‘normal’ (negative for RHD) when the age was ≤20 years and in ‘definite’ and ‘normal’ cases for people aged over 20 years.

Cases identified as ‘definite’ and ‘borderline’ were referred to the NIHMP cardiology out-patient clinic, where a cardiologist with echocardiographic expertise repeated the echocardiographic investigation using the fixed ultrasound echocardiography machine Siemens “Acuson X700”, which is known to have a better and higher resolution, alongside a baseline clinic cardiac evaluation and electrocardiogram (ECG). The ECG was performed with “Mortara ELI 230” compact device. The prescription of secondary prophylaxis was recommended and initiated for the confirmed cases of ‘definite RHD’, consisting in a single intramuscular injection of benzathine penicillin G every 3–4 weeks (0.6 million U < 20 Kg; 1.2 million U > 20 kg until the age of 21 years for case of mild RHD (silent, asymptomatic cases), according to the RHD Australia and New Zealand Society Guidelines, 2012 [19].

### Imaging modality

WHF guidelines for diagnosis of RHD were followed. An experienced cardiologist performed standard 2D transthoracic echocardiogram with a portable machine (Vivid e, GE Healthcare 2007) on site with a 1.5- to 2.5 MHz transducer 3S-RS. Before scanning, machine was adjusted with settings proposed by WHF guidelines: Nyquist limits for color-Doppler echocardiography set on maximum to avoid overestimation of jet length; images for assessment of valve and chordal thickness were acquired with harmonics turned off and probes with variable frequency set on ≥2.0 MHz; gain settings was adjusted to get the best resolution; other settings (including depth, sector size, and focus) were optimized to obtained maximal frame rate (between 30 and 60 frames per second) and images resolution.

### Data entry and statistical analysis

All data were double-entered into an Excel database and cross-checked for validity. Each participant was anonymized and identified by a 4-digit code.

Data was analyzed in Stata (version 14, StataCorp, College Station, Texas). Descriptive statistics were used to map the different characteristics. Bivariate analysis was performed on pooled data to identify participant characteristics associated with positive RHD cases identified by echocardiography, by using Pearson’s Chi-squared test or Fisher’s exact test, as appropriate. Risk factors independently associated with RHD were identified through a multivariate logistic regression model. Independent categorical variables were screened for multi-collinearity.

### Ethics approval

The study was approved by the Ethics Review Board of “Fondazione PTV Policlinico Tor Vergata” (Rome 2 University Hospital), on the 17 January 2016, protocol number 227/16. Permission to entry in Reception Centers for unaccompanied foreign minors was obtained from the Minors’ Protection Operational Unit of Rome Municipal Social Policies Department.

## Results

### Study population

From February 2016 to January 2018, 653 individuals were screened (Table 1): 639 were males (97.9%) and the mean age was 16.4 years (range 11–26) with 624 individuals being less than 18 years old (95.6%) (Table 1 and Table 2). The majority of the people screened were from North Africa (442, 67.7%), in particular from Egypt (422), followed by West Africa (136, 20.8%), East Africa (49, 7.5%) and South Asia (26, 4.0%). The proportion of the screened migrants presenting with a previous history of fever/sore-throat was 65.2% (426), while only 22.4% reported to have National Health System (NHS) coverage and access (146) (Table 2).

**Table 1:** Countries of origin of the screened individuals

Characteristics	Total	Proportion
Egypt	422	64.65%
Eritrea	39	6.0%
Gambia	33	5.0%
Nigeria	28	4.3%
Guinea	19	2.9%
Ivory Coast	19	2.9%
Senegal	17	2.6%
Bangladesh	13	2.0%
Mali	10	1.5%
Tunisia	9	1.4%
Morocco	9	1.4%
Pakistan	8	1.2%
Somalia	7	1.1%
Afghanistan	6	0.9%
Ghana	5	0.8%
Sierra Leone	2	0.3%
Cameroon	1	0.15%
Chad	1	0.15%
Ethiopia	1	0.15%
Mauritania	1	0.15%
Sudan	1	0.15%
Tanzania	1	0.15%
Turkey	1	0.15%

**Table 2:** Baseline characteristics of the screened individuals

Characteristics	Total	Proportion/Range
Number of screened individuals (n)	653	
Age in years, mean (SD)	16.4 (1.3)	11–26
Male	639	97.9%
Female	14	2.1%
Age Category		
• < 12	1	0.2%
• 12–15	111	17.0%
• 16–18	527	81.7%
• > 19	14	2.1%
Geographic Area of Origin		
• North Africa^a^	442	67.7%
• West Africa	136	20.8%
• East Africa	49	7.5%
• South Asia	26	4.0%
Previous History of Fever/Sore-Throat		
• Yes	426	65.2%
• No	227	34.8%
NHS Coverage^b^		
• Yes	146	22.4%
• No	507	77.6%
Previous Hospitalization		
• Yes	8	1.2%
• No	645	98.8%
Detection of Systolic Murmur		
• Yes	195	29.9%
• No	458	70.1%

^a^Egypt is the country most represented with 422 screened individuals

^b^NHS: National Health System

Among the 653 screened individuals, 6 ‘definite RHD’ were identified, yielding a detection rate of 9.2‰ (95% CI 4.1–20.3), while 285 (436.4‰) were defined as ‘borderline RHD’ (95% CI 398.8–474.9). Among the 285 individuals to be retested 113 were lost in follow-up (39.6%). There was no significant difference, in terms of age, place of origin or valve lesion, between the individuals who were lost to follow up and the ones who were still available for the second test.

Systolic murmur was identified in the 29.9% of the total screened population (195/653). The systolic murmur was associated, in few cases (5), with a diastolic component linked to aortic valve regurgitation.

Out of 172 ‘non-negative, bordeline’ cases available and retested, additional 11 were categorized as ‘definite RHD’ (prevalence of 26.0‰ [17/653]; 95% CI 16.2–41.5‰), while 122 were confirmed as ‘borderline RHD’ (prevalence 186.8‰ [122/653]; 95% CI 158.7–218.7‰) (Fig. 1, Table 3).

**Table 3:** Characteristics of valve disease in 139 children diagnosed with latent RHD. The combinations of cardiac findings at the echo-cardio examination showed in the table, define and differentiate the cases of ‘definite’ and ‘borderline’ RH, according to the World Heart Federation criteria [18]

	Characteristic of the MV valve	Number	Proportion
Definite RDH		17	
Presence of pathological MR^a^ with at least two morphological features of RHD of the MV^b^	Thick AMVL+ Thick Chorde	8	47.0%
	Thick AMVL+ Thick Chorde+ Excessive Leaflet Motion	6	35.3%
	Thick AMVL+ Thick Chorde+ Restricted Leaflet Motion	2	11.8%
Multiple valve lesion		1	5.9%
Borderline RHD		122	
	Thick AMVL + Thick Chorde	72	59.0%
	Thick AMVL + Thick Chorde+ Excessive Leaflet Motion	32	26.2%
	Thick AMVL + Thick Chorde+ Restrictive Leaflet Motion	10	8.2%
	Others	8	6.6%

^a^MR: mitral regurgitation

^b^MV: mitral valve

**AMVL: anterior mitral valve leaflet

Among the Egyptian population who represented the majority of the individuals assessed, 8 cases of ‘definite RHD’ (8/422) and 78 cases of ‘borderline RHD’ were identified, yielding a detection rate of 19.0‰ and 184.8‰, respectively.

The age group with the highest detection rate for ‘borderline RHD’ was 12–15 years with a prevalence of 225.2‰, while the age group 16–18 years had the highest detection value for ‘definite RHD’ (28.5‰) (Table 4).

**Table 4:** Prevalence of latent RHD and Bivariate and Multivariate Logistic Regression Analysis to assess risk factors associated with latent RHD (analysis after retest of the positive screening)

	Number	*Prevalence ‰	Multivariate Analysis *P* value	Odds Ratio (CI 95%)
**Borderline RHD**				
Total	122/653	186.8‰		
Age Category				
• < 12	0/1	0		
• 12–15	25/111	225.2‰	0.2	
• 16–18	95/527	180.3‰	0.2	
• > 19	2/14	142.9‰		
Origin				
• North Africa	79/442	178.7‰	0.9	
• West Africa	29/136	213.2‰	0.6	
• East Africa	10/49	204.1‰	0.9	
• South Asia	4/26	153.8‰	0.5	
Systolic Murmur among Borderline RHD	74		< 0.001	4.3 (2.8-6.5)
NHS Coverage among Borderline RHD	36		0.2	
Past history of Fever/Sore Throat among Borderline RHD	85		0.3	
Past Hospitalization among Borderline RHD	1		0.3	
**Definite RHD**				
Total	17/653	26.0‰		
Age category				
• < 12	0/1	0		
• 12–15	2/111	18.0‰	0.3	
• 16–18	15/527	28.5‰		
• > 19	0/14	0		
Origin				
• North Africa	9/442	20.4‰	0.8	
• West Africa	5/136	36.7‰	0.5	
• East Africa	1/49	20.4‰	0.9	
• South Asia	2/26	76.9‰	0.1	
Systolic Murmur among Definite RHD	12		< 0.001	5.2 (1.7-15.2)
NHS Coverage among Definite RHD	5		0.9	
Past history of Fever/sore throat among Definite RHD	10		0.8	
Past Hospitalization among Definite RHD	0		0.1	

The number of ‘definite RHD’ cases detected through the second and more accurate test (*n* = 17), was 3-fold higher than those detected by screening (*n* = 6). The most frequent morphological feature of pathological mitral valve was the ‘thick anterior mitral valve leaflet plus thick corde’ combination present in 47% (8 cases) and 59% (72 cases) of confirmed ‘definite’ and ‘borderline RHD’, respectively (Table 2).

Two cases of ‘incidentaloma’ were identified and immediately referred to the Cardiology Unit of the Rome 2 University Hospital. One was an abnormal aortic dilatation with aortic regurgitation in a bicuspid aortic valve, which was successfully treated with cardiac surgery. The second one was an atrial septal defect with significant inter-atrial shunt causing pulmonary arterial hypertension, which underwent percutaneous implantation of device closure.

### Risk factors associated with latent rheumatic hearth disease

Gender was excluded because of collinearity with the dependent variable (presence of both ‘definite’ and ‘borderline RHD’). Age group, previous history of sore throat/fever, NHS coverage and previous hospitalization were not associated with increased odds of latent RHD.

The presence of systolic murmur was a strong predictor for both ‘borderline’ (OR 4.3 [2.8–6.5]) and ‘definite RHD’ (OR 5.2 [1.7–15.2]) (Table 4). The positive predictive value (PPV) of systolic murmur valve for detecting, at screening, any form of subclinical RHD was 80.5% (95% CI 74.1–85.6), while the negative predictive value (NPV) was 70.7% (95% CI 66.3–74.8).

## Discussion

In this study, we report on the retrospective analysis of an innovative screening strategy for the detection of RHD in Italy, a high-income country that, in recent decades, has experienced a significant decrease of the burden of RHD [4]. This strategy is based on a ultrasound-based active case finding approach among specific, discrete populations considered at high risk for RHD, by providing screening opportunities to unaccompanied foreign individuals (the majority of whom are minors) coming from low-income countries and sheltered in centers and family houses in Rome city. Our results illustrate the feasibility of this approach, reaching more than 650 individuals over 23 months of operations and leading to the identification of asymptomatic cases of RHD.

A number of key observations and possible recommendations are highlighted by this study. First, the screening activity, coupled with a second more in-depth and thorough examination of the ‘non negative’ cases, performed by expert cardiologists using a fixed ultrasound echocardiography machine, was able to yield a case detection rate for latent ‘definite RHD’ above 20‰ and similar to the one described in the literature [5–7] (including the recent paper from Kotit et al., describing the echocardiographic screening-based prevalence of RHD disease among Egyptian school children [8]).

The fact that RHD almost disappeared in developed nations has led to a generalized neglect of the disease in recent years: the results of our study calls for reviving its medical education, attention and vigilance, as medical doctors and health systems have become unfamiliar with the condition.

Second, the presence of a systolic murmur was strongly predictive of ‘definite RHD’ (*p* < 0.001 and OR of 5.2 [CI 95% (1.7–15.2]). Twelve out of 17 ‘definite RHD’ were associated with a systolic murmur. The same significant association was observed among those showing a ‘borderline RHD’. However, while its PPV was > 80%, the 70.7% NPV of the valve murmur cannot be considered a stand-alone clinical criterion for defining/selecting patients who will access second-level cardiac investigation, as this will equate to under-diagnosis (missed cases) and under-treatment in roughly more than one third of patients who do not present systolic murmur. Thus, the findings of this study represent an additional element supporting the recommendation of systematically providing echocardiographic investigation among the vulnerable population of migrant/refugee children, irrespective of the presence of a valve murmur.

Third, the combined screening and retesting activities identified a surprisingly and unexpected high number of ‘borderline RHD’. To our knowledge, this is the first time that a detection rate for ‘borderline RHD’ scores around the value of 200‰. The justification for such a result can be found in a) the demographic nature of the targeted group, composed by a group of people at very high risk of developing RHD (on top of the classic hardship experienced by any migrant/refugee, the unaccompanied foreign minor usually shares the additional burden of the lack of family care, potentially compounding the already precarious general living conditions). Consensus is emerging that this group of people with asymptomatic RHD should be considered for secondary antibiotic prophylaxis with benzathine penicillin G (BPG) to prevent progression to symptomatic disease [20, 21]. As the adherence to BPG prophylaxis is poor worldwide (less than 50%), with the exception of some developed countries, such as New Zealand and Samoa [22], it would be important to understand who can really benefit from such preventive approach. There is limited information on the natural history and virtually no evidence upon which to base the management of children and adolescents with asymptomatic subclinical rheumatic heart disease that is detected on active surveillance. The time has come to launch large-scale prospective studies of the natural history of latent rheumatic heart disease and trials to assess the efficacy of penicillin prophylaxis to prevent progression of disease [21].

In recent years, some research groups have shared their findings on preliminary results on the evolution of ‘borderline RHD’, by performing short and medium-term follow-up studies [23–28]. Most of these longitudinal investigations consistently showed that around 10–15% of ‘borderline’ disease progressed into definite disease.

All these elements point to the responsibility for the health system of the rich countries to do more in understanding both the disease progression and optimal ways to identify RHD at an early stage to prevent its worsening.

Although much work remains to be done, particularly in regard to promoting adherence to secondary prophylaxis, the cost-effective argument for a secondary prevention remains strong [29–31], in view also of the potential loss of economic productivity from a disease that ‘cuts young people down in their prime’ [13], especially in rich settings where the epidemiological transition means that a relevant part of the current and future workforce is composed and will be sustained by recent/future migratory waves.

As Marijon warns [32], ‘“the affluent world cannot afford complacency; large population movements and refugee crises can displace persons with rheumatic heart disease to developed nations”. RHD is not restricted to low-income or tropical countries, but it is also a matter of potential concern in middle income and highly developed countries. The refugee crisis in Europe testifies this phenomenon and the findings of our study support the call to arms issued by some authors in fostering attention in rich countries [33].

The study faced a number of limitations. Mainly, it was led by the international NGO MSF, prompted by a strong determination and advocating spirit (along with financial, logistic and human resources investment), which may not reflect the political will of the health systems of rich countries. Moreover, the study used a portable machine which had an imaging resolution lower than the non-portable, fixed machine used in the ‘re-testing stage’. This implied that, during the ‘screening stage’, our measurements, especially on mitral valve leaflets thickening, could have been underestimated. Finally, the strategy put in place was not able to re-assess/retest all the initially screened ‘borderline’ cases, as 113 of them were lost on follow-up, having abandoned the official reception circuit for different reasons (e.g. become adults and no longer traceable; transferred to other Italian Regions; unavailable and or unwilling to receive a the second control). Although MSF employed in its team a cultural mediator, the lack of a preliminary and constant sensitization among the target population, as well as the absence of an established community network of social workers able to trace back the defaulters may have contributed in determining such high rates of lost in follow-up.

The major strength of this study laid on the commendable example of synergy and collaboration between an international non-governmental organization (MSF) and national academic institutions, which can become a source of inspiration and replication for other actors in similar or different settings.

## Conclusions

The screening for RHD among the unaccompanied migrant minors in Italy proved to be feasible. The burden of ‘definite RHD’ was similar to that identified in resource-poor settings, while the prevalence of ‘borderline’ cases was higher than reported elsewhere. In view of these findings, and of the fact that protecting promoting the health and wellbeing of migrants is high on the agenda of the WHO [34], the health system of high-income countries, hosting migrants, refugee and asylum seekers, are urged to adopt screening of such populations, especially for the silent and marginalized population of refugee and migrant children, often arriving in Europe unaccompanied and defenseless.

## Abbreviations

AMVL: Anterior Mitral Valve Leaflet
HIC: High Income Country
LIC: Low Income Country
MIC: Medium Income Country
MR: Mitral Regurgitation
MSF: Médecins Sans Frontières
MV: Mitral Valve
NHS: National Health System
NIHMP: National Institute for Health, Migration and Poverty
RF: Rheumatic Fever
RHD: Rheumatic Heart Disease
WHA: World Health Assembly
WHF: World Heart Federation
WHO: World Health Organization
